# Informal settlements, Covid-19 and sex workers in Kenya

**DOI:** 10.1177/00420980211044628

**Published:** 2021-10-07

**Authors:** Rahma Hassan, Teela Sanders, Susan Gichuna, Rosie Campbell, Mercy Mutonyi, Peninah Mwangi

**Affiliations:** University of Copenhagen, Denmark; University of Leicester, UK; University of Nairobi, Kenya; University of Leicester, UK; Bar Hostess Empowerment and Support Programme, Kenya; Bar Hostess Empowerment and Support Programme, Kenya

**Keywords:** gender violence, informal settlements, Nairobi, poverty, sex work

## Abstract

This paper highlights the challenges faced by female sex workers living and working in the urban informal settlements in Nairobi, Kenya, during the Covid-19 outbreak and the aftermath of the pandemic. Using data collected through phone interviews during the immediate crisis, we document the experiences of urban poor sex workers, illustrating the acute problems they faced, including precarious housing with the reality of eviction and demolition. The paper highlights the ramifications of the Covid-19 crisis for the sex industry and predominantly women working within this informal, illegal economy. Through our empirical data we illustrate how the nature of selling sex has changed for sex workers in this context, increasing risks of violence including police abuses. We argue that examining the Covid-19 crisis through the lens of one the most marginalised populations graphically highlights how the pandemic has and will continue to deepen pre-existing structural urban inequalities and worsen public health outcomes among the urban poor. Sex worker communities are often located at the intersections of structural inequalities of gender, class, race and nation and the socio-spatial fragmentations of how they live make them some of the most vulnerable in society. We close with comments in relation to sexual citizenship, exclusionary state practices and the feminisation of urban poverty.

## 摘要

本文重点介绍了在新冠肺炎疫情期间和疫情之后，在肯尼亚内罗毕城市非正规住区生活和工作的女性性工作者所面临的挑战。我们使用在紧急危机期间通过电话采访收集的数据，记录了城市贫困性工作者的经历，说明了他们面临的严重问题，包括不稳定的住房以及被驱逐和拆除的现实。本文强调新冠肺炎危机对性行业以及在这种非正规、非法经济中工作的人们（主要是女性）的影响。通过我们的经验数据，我们说明了在这种环境下，性工作者的性出卖的性质所发生的变化，这些变化增加了包括警察滥权在内的暴力风险。我们认为，通过一个最边缘化的人群的视角来审视新冠肺炎危机，能够生动地凸显疫情如何已经并将继续加深本已存在的结构性城市不平等，并导致城市贫民公共卫生状况的恶化。性工作者社区通常属于遭受性别、阶级、种族和民族等结构性不平等多重影响的人群，生活中的社会空间碎片化使他们成为了社会中最脆弱的群体。在文章的结尾，我们发表了性公民身份、排斥性政府行为和城市贫困女性化方面的评论。

## Introduction

The Lancet HIV (2020), early in the global Covid-19 pandemic, cautioned that certain populations would be at major risk of not only contracting the virus but of consequential health, social and economic disasters. Sex workers, homeless and transgender populations were all named as populations who would be hit hardest by the pandemic, government restrictions and broader implications (see Amnesty International, 2020; Ataguba, 2020; Care International, 2020; UNAIDS, 2020). This paper aims to highlight the specific circumstances and consequences for sex workers living and working in informal settlements during the Covid-19 pandemic in the urban city of Nairobi, Kenya. We highlight the inequalities surrounding their lives and the extreme effects brought about by the disease and associated government policies on movement restrictions. We note specifically how sex workers had to adapt their sex work, the longer-term impacts on the nature of sex work and the new and sometimes heightened risks that came along with this change. During 2020 there was an extensive and unusual global media focus on sex workers and the effects of the pandemic, which we have analysed with a focus on Africa (Campbell et al., 2020). This attention was partly a continuation of a long term sensationalisation of sex work/ers in the media and perpetual moral panic over their bodies and trafficking, ideas around disease/infection and their ‘deviant’ status (Weitzer, 2006). Academic and community research established that sex workers have been further excluded from health care during the crisis (Howard, 2020; ICRSE, 2021; ICRSE and SWAN, 2020; Platt et al., 2020; Sanders and Hassan, 2021; Shareck et al., 2021), and we have discussed health care access implications in a separate paper (Gichuna et al., 2020). A spike in the number of sex workers who have experienced violence in Kenya has been reported during the pandemic (Bhalla, 2020). This has been reported to include gender-based violence from a client or regular non-paying sex partner including being short-changed by clients; refusing to pay the agreed fee, or not paid at all (Bradbury-Jones and Isham, 2020). These scenarios, where negotiated consent for commercial sex is breached are indeed rape for sex workers. Our paper brings into focus the longer-term impacts on selling sexual services, gender-based violence and the lack of protection for sex workers in the Nairobi context experience.

One important context to the lives of sex workers in Nairobi is the informal settlements and subsequent demolition during the Covid crisis. The majority of sex workers in Nairobi live and work in informal settlements (Amuyunzu-Nyamongo et al., 2007; Atuahene, 2004). Previous research by this team has found a large proportion of sex workers living in Kariobangi and Mathare areas with others living in neighbouring informal settlements (Hassan et al., 2018). Mathare slums^1^ are groups of informal settlements known for their dense population while Kariobangi is a low-income residential area with a mix of slums and low-quality urban apartments (Austrian et al., 2020). The demolition of dwellings in Kariobangi that took place on 4th May 2020^2^ during the early days of the pandemic serves as an example of the unresolved contestation over land rights and the powerlessness of the urban poor in the informal settlements. An estimated 8000 people occupied the dwellings and were left homeless amid the crisis and restrictions on movement.

This study only included sex workers living in Nairobi East but the population of sex workers experiencing the issues made visible by our research is much larger, considering that sex workers live and work in other informal settlements in Nairobi. For sex workers, informal settlements are not just homes but house the ‘hot spots’– the bars, clubs, restaurants and brothels where sex workers meet customers and carry out their business. In this study, we seek to understand the lives of sex workers living and working in informal settlements in Nairobi during the Covid-19 pandemic and the aftermath of their lives and work after the demolitions. This paper is based on data from the first research study to collect empirical data directly from Nairobi’s sex workers during the crisis, other published material has drawn on observational data from health care workers working with sex workers in Nairobi (Kimani et al., 2020). This article aims to contribute not only to the emergent global literature on the impact of Covid-19 on sex worker populations but more widely to the literature on sex workers living and working in informal settlements.

## The study: Materials and methods

This study is based on a partnership between researchers at the University of Leicester (from which ethics approval was gained) and the Nairobi based third sector organisation, the Bar Hostess Empowerment and Support Programme (BHESP). BHESP is an organisation for and run by sex workers, women who have sex with women, women using drugs and bar hostesses in Kenya.^3^ This collaboration had previously carried out participatory research, the community based and peer lead nature of BHESP, with strong established networks of sex workers in Nairobi’s informal settlements, experienced researchers on the ground and workers providing support services throughout the crisis. They were all vital in enabling this study to take place rapidly at a time when sex workers were in crisis. The study conducted in Nairobi East focussed on sex workers who were BHESP service users living in the informal settlement areas neighbouring Kariobangi, Kasarani and Jogoo Road.

All the data collection was conducted digitally through telephone interviews, as face to face recruitment and interviews were not possible due to Covid-19 restrictions on social contact and movement. Respondents were initially informed about the study through a text message seeking consent to participate, after which the best time to conduct the interview was established. Measures to ensure participant confidentiality were observed with participant identification details recorded separately from the data collected. Participants were further given information about the study and their voluntary participation including assurance of confidentiality. Verbal consent was always sought, and participants were free to discontinue the interview at any time. The study relied on phone interviews but sometimes selected participants could not be contacted by phone, so we also adjusted our method to accommodate sex workers who shared phones to ensure we reached a broad sample. This was done by calling and tracing the respondent and scheduling interviews based on their ability to use the shared phone without breaching confidentiality. Other respondents expressed phone network challenges, and, in some instances, participants had not charged their phones due to the disruptions and unavailability of electricity in the informal settlements. The study team adjusted to these challenges by replacing the participants who were unavailable through the database as well as arranging for interview times and schedule to enable the participants to make time for the interview in a safe environment. Respondents were also facilitated with money to buy airtime to call back or respond to the questions on chat if required to do so.

A total of 117 sex workers and 15 health care practitioners were interviewed between May and June 2020.

Participants were asked about their experiences of working during the pandemic and the challenges they were facing as sex workers. Health practitioners were asked about access to health care and practical solutions to the crisis for this population. Data collected was recorded in MS Excel and grouped according to the questions posed during the study. The project team held regular Skype meetings to discuss findings as they emerged to understand the data and develop collective interpretation. The data collection was terminated once the team identified patterns of saturation and no new data was being gathered. The data was analysed through thematic analysis and key themes and sub themes identified. After the data collection in 2020, during Spring/Summer 2021, observations were made by the fieldwork team in relation to the lifting of government restrictions and then the imposition of another lockdown. Informal conversations were held between peers in the research team who volunteered with BHESP to reflect on the longer-term changes to work and livelihoods. At the time of writing Nairobi had been returned to full lockdown status and the government mobility restrictions and business closures were reinstated. This movement in and out of various levels of government restrictions is likely to characterise the city for some time to come.

## Findings: (Not) selling sex during a pandemic

Sex workers immediately suffered an acute loss of income when government mobility restrictions were imposed on the city because they were separated from clients. Previously it was rare for clients to meet sex workers in their homes. Sex workers described how their clients were reluctant to come to their homes and how they were unable to visit their clients’ homes due to travel difficulties, costs and safety concerns:

*I am not able to host clients at my home at Mathare. Most of the clients have fear of coming to our homes. I am also afraid of going to meet them in their houses as it feels unsafe. Some live across the city and I didn’t have money to pay for transport to reach them. It is getting extremely hard to meet clients and most times the discussion on phone is about getting very few clients and they are not paying well. (Kasarani, 18 years old*)

With the virus passing easily through close contact, sex workers found it very difficult to implement social distancing measures and many clients were fearful of transmission. The decline of customers wanting to buy sex was dramatic. One of the respondents explained:

*Most people are afraid of contracting Covid-19……… With the bars and hotspots closed, I now rely on clients who I can call but still, they are also not willing to meet me. When I call them, some say they are afraid of contracting Covid and they would not want to risk their lives and that of their families by meeting me. Clients do not listen even when we explain that we are observing the measures not to contract the disease. (Kasarani, 26 years old)*

Social distancing and stay-at-home rules meant that most of the entertainment places (where women met their customers) had closed. We spoke to sex workers who could no longer meet the needs of their families because of the disruption to their work:

*The situation is bad. I am not able to earn a living now that there are so many restrictions affecting our sex work. We are not getting clients because we cannot go to our hotspots and clubs. I have not paid rent for the last 2 months and we are not getting enough food. I am lucky schools are closed; I could not have managed to raise the school fees. They are supposed to be studying online especially one who is a primary school candidate, but I cannot afford the TV subscriptions. (Kariobangi, 29 years old)*

Respondents reported that the clients were also facing financial challenges and therefore they could either not afford to pay for sex or they would negotiate to pay less:

*Before the curfew was introduced, in a week I would make between 70 dollars to 100 dollars and I would work about 5-6 days. Currently, it is extremely hard to make 50 dollars a week. We are not able to meet clients as everything is closed, and the clients are also affected so they tell us that they have also lost their jobs or that they are unable to make extra money to pay. (Kariobangi, 27 years old)*

For sex workers, the challenges of meeting clients during the disruption of leisure spaces also related to their housing conditions within the settlements. Informal settlements lack adequate housing facilities, dwellings are often one room for the whole family. This presents another barrier for sex workers, who did not have the option of meeting clients at home. This restricts their opportunities for income compared to sex workers who have more adequate housing with private spaces to meet their clients. The housing situation was further complicated by the closure of schools which meant children were at home. The experience of one of the respondents captures the predicament:

*I am suffering because it now is too hard to do my job. We cannot go to the hotspots at night because of the curfew. I was living in Kariobangi, but our houses were demolished. I was living with three of my children and one of my sisters. I had to take two of the children to my mother because I am not able to take care of all of them. (Kariobangi, 22 years old)*

Another respondent described the difficulty of doing sex work presented by the limited housing space including the consideration that most schools were closed for several months:

*I am finding it extremely hard to work during the day, clients are very few. I also do not have the space to do my sex work, children are at home all day, so I cannot take clients to my house. (Kariobangi, 30 year old)*

The matter of land tenure in the urban informal settlements created precarity for residents. The situation in Kariobangi with demolition and forced evictions dramatically affected sex workers, who became destitute with nowhere to live during the lockdown. Many respondents described how both their income and housing had disappeared:

*My situation is bad. I used to stay in Kariobangi, but our houses were demolished. I have since moved in with a friend, but I do not feel comfortable here because the space is small, and it is hard for her to host as well. I feel I am a bother to her because I have a child and I am currently pregnant and almost giving birth. I am suffering so much; in my condition, I cannot get clients. I feel Covid-19 came with loads of misfortunes for me because so many of my friends who could help me are also affected by the demolitions of houses. (Kariobangi, 27 years old)*

Most of the sex workers we spoke to expressed how difficult it was to work within the curfew and cessation of movement:

*I feel we are just stuck with so many problems. There is no work, we have to get home early because of the night curfew. Things are not good. I hardly get clients nowadays, yet I still need to feed my children. We just stay in the ‘plot’ (at home) all day with no work. We are just suffering and struggling to pay rent and feed ourselves. (Jogoo Road, 32 years old)*

With the cessation of movement measures put in place to curb Covid-19 from spreading to different parts of the country, sex workers were cut off from their clients who live outside Nairobi during the immediate restrictions in 2020. The lucrative form of overnight stays out of town had stopped and there was no prospect of this starting up again. One of our respondents explained:

*I had occasional trips outside Nairobi sometimes I would be away with a client for a week and that would be enough to address my household needs for the month. Most of the clients used to prefer to go out of town and take us to accompany them to holiday destinations like Mombasa. Since the Covid-19 restrictions of movement were announced I have not received any call for the trips. (Kasarani, 23 years old)*

Bars in Kenya were closed continually for seven months (March to September 2020). Once they were reopened the restrictions at night continued which affected sex workers who work mostly at night. This meant that the trading hours remained restricted with bars expected to close at 10* *pm. The announcement in March 2021 of renewed lockdown and cessation of movement measures issued by the government, that included full closure of bars and restaurants, implied that challenges for sex workers were not temporary with little prospect of returning to pre-pandemic conditions.^4^

## No income, no alternative work, no protections

For the women, the struggle in informal settlements was to earn enough money through selling sex so they could eat for the day and meet the basic requirements of their family (food, medicine, transport costs and rent) (see Madhavan et al., 2020 on food insecurity amongst women in Nairobi). We discuss the economic and social effects of Covid-19 for sex workers which include deteriorating or complete loss of income, lack of alternative income, exploitation and stigma. Most of the respondents reported that their income had been reduced significantly and that they could no longer survive with their current earnings. These quotes were typical from all 117 respondents:

*We can’t go anywhere now because we are being asked to stay at home. So that has meant that our lives have changed drastically, and we do not have basic needs. We are staying at home all the time and that means we do not have any money coming in to support our families. Now I am running out of food and I have no money left. With no clients to go to we do not have an income. (Kariobangi, 46 years old)*

*In the past sometimes during the weekend I would earn about ksh 1000 but now I get between ksh 100-500 in a whole week. It is frustrating because by the time I get a single client I have to really beg and convince them. Sometimes I ask friends to connect me to possible clients because it has become so hard to find them. (Kasarani, 22 years old)*

*Previously I could earn 50 dollars per day but now it is as low as 5 dollars on a good day. Our clients are now stuck at home as well with their families and even those who would meet us at night are restricted by the curfew. (Kasarani, 34 years old)*

One of the pressing economic challenges we found among sex workers is the lack of alternative income. Africa was already in the grip of a significant youth unemployment problem when the pandemic hit (Filmer and Fox, 2014). Research on young female sex workers by UNAIDS (2014) found that 40% of female sex workers started selling sex before the age of 18. The lack of opportunities was stark for this group of women:

*I have not found another job. Getting a job these days is difficult, there are no jobs. The alternative job I would be interested in is working in a club but now I cannot find it as they are closed. (Kariobangi, 26 years old)*

*I do not have any other source of income. I have no idea what other job I can do because I am so used to my sex work. I cannot even start a business because it needs capital and that is what I do not have at the moment. (Kariobangi, 25 years old)*

*Many people expect us to stop working as sex workers and get other jobs, but getting a job in Kenya is so hard for everyone we have no alternatives. (Kasarani, 30 years old)*

The levels of desperation were incredibly high, with no alternative employment and no government protections for sex workers to fall back on. There was a continual sense that other disadvantaged groups were prioritised:

*I have not got any assistance, I just see on TV people getting assistance. I heard the government is employing youths for manual jobs in the areas of residence but I have not got any. In April I was able to pay rent using my savings but this month I have not paid, I might be locked out if the landlord comes demanding for the cash. (Kariobangi, 23 years old)*

*I have not received any help so I survive with the little I am getting from sex work*.

*The government is providing some donations but I have not received any. As sex workers, we are discriminated against. I cannot go to queue for the donations or register, I do not want to get insults from my harsh neighbours, especially women (Kariobangi, 29 years old).*

There was a consistent concern that they would be identified as a sex worker and start receiving harassment and possible violence from others in the neighbourhood. The levels of stigma attached to sex work compounded the issues of accessing protections that should have been available for all:

*We are not benefiting from the support being given by the government. There is a lot of discrimination and corruption by those distributing the aid. My neighbours especially women are hostile towards me because they know I am a sex worker. They cannot include me in the list of the needy ones. (Kariobangi, 25 years old)*

*I have given my name in the many lists going around for food donations but I have not received anything. I feel we are being discriminated against. We are about 6 sex workers in residence and none of us have received anything. The government has been hiring youths for cleaning jobs in the residences but none of us got the jobs. (Kariobangi, 35 years old)*

The Covid context and specifically the lack of income were one set of issues. There were additional enhanced harms and risks that participants relayed, particularly in relation to violence from a multitude of sources. Below we discuss some of the findings in relation to violence from clients, harassment from police and broader gender-based violence to which they were often victims.

## Harms: Risks from clients, police and gender-based violence

One of the outcomes of the government restrictions was that many women were at home with their abusers and were unable to access support services due to the restrictions on movement (United Nations Development Program, 2020). Christian Aid (2020) reported that as the number of coronavirus cases continued to rise in Nairobi, the number of women and girls facing violence continued to increase. In the year 2020 BHESP received reports of violence from 2733 service users. This is a threefold increase compared to the previous year. Most of the violence reported was perpetrated by clients or partners at home. Sex workers were under increased pressure to meet clients in customer homes as the bars and brothels were closed, putting them more at risk of violence as described by the following respondent:

*Some sex workers are now meeting clients at home, but this is something we have been avoiding. Many of the instances of violence have been from people who went to client’s houses. At the hotspots, we feel safer and we can always ask a friend to be on the lookout. In some instances, we take the number plate of the clients and report them. Being with them at their homes puts a lot of us at risk because we are alone and vulnerable. We also are now not together with our colleagues to give each other support and even share our fears or challenges with clients. The clients also are afraid of Covid-19 and given many truck drivers have tested positive for Covid-19 many of us are likely to get affected which is going to affect our work. (Kasarani, 30 years old)*

The above quote confirms much of the literature on safety in relation to working conditions and sex work – where there is isolation, secrecy and no collective setting there are increased risks and harms (Kinnell, 2008; Sanders, 2016). Respondents confirmed that not only are they at risk of infection in the context of Covid-19 but they are at risk of harm from clients. The relative safety afforded by previous working conditions had gone:

*The risks are rising especially now that we are not going to our hotspots. Clients are calling sex workers to their houses and taking advantage of them. At the hotspots it is safer because we stay in groups so once you get a client the other sex workers get to know your whereabouts and who you have left with There is a lady who recently went to a client’s house. The client had 2 other men in his house so they took rounds with her, molested her and threw her out without pay. It was very humiliating and painful. (Kariobangi, 25 years old)*

The women recounted stories of violence to each other and warned each other of concerns about individuals. We have reported previously on the impact of digital technologies on sex work and the innovations in relation to how sex workers use messaging services to contact each other (Campbell et al., 2019). This certainly is the case in Nairobi where a vibrant online community exists to warn and support each other. This has been essential for the community over the pandemic period as several murders have occurred:

*We have been talking and sharing among ourselves as sex workers and cautioning each other, some of us are even being killed. A lady we know was molested, stabbed and left for the dead by a client. He escaped but luckily he took her phone instead of his and police managed to trace and arrest him. They are really mistreating us. (Kariobangi, 31 years old)*

We found in this study that sex workers changed their working practices by offering reduced rates for services, resulting in reduced negotiating power with the few customers that were accessible:

*We are no longer able to negotiate and get what we feel is worth our services. Sometimes clients will claim that they can only afford 10 dollars which is little but since I have no alternative, I just must take it. I feel they are also taking advantage of the situation. I recently had a client whom we agreed he would pay me 50 dollars but after the services, he only paid me 20 dollars. I had to take it. (Kasarani, 25 years old)*

The fact that supply of sex workers far outweighs the demand from clients gives the buyer the greater power, especially in a context where sex work is criminalised. We know from a metastudy of the impact of criminalisation on sex workers, that client violence is enabled by the framework of illegality (Platt et al., 2018). Sex workers in Nairobi discussed how their weak economic situation had been made increasingly dangerous in the pandemic:

*I used to get like 6 clients in a day, now even 2 are hard to get. The pay is very poor, whatever the client offers is what you take. I used to take home around ksh 5000 in a day, the highest I make now is ksh 1000 or 1500 on the higher side. I cannot demand a higher pay. I have to understand the client’s situation as well, their finances have also deteriorated. I accept what they can afford. (Kariobangi, 22 years old)*

Alongside the gender-based violence from clients, we found sex workers experienced physical assault and various forms of exploitation from the police such as bribery and extortion. We know that police brutality is not uncommon against sex workers where sex work is illegal (Crago et al., 2008; Dewey and St Germain, 2014) but given the heightened patrols by the police during the lockdown hours and the curfew at night, sex workers who encountered the police reported being beaten or having to pay bribes to make it home safely. Sex workers revealed:

*Police have become our greatest enemies; it doesn’t feel safe anymore going to the police station to report a case. They are always asking for bribes and failure to provide bribes will result in illegal arrest. The police are also taking advantage of us in case they find you outside during curfew time the ask for sex in return for your release. (Jogoo Road, 26 years old)*

*Police are taking advantage of the situation. If they find you in the wrong you have to bribe them or else they arrest you and take you to quarantine. Some are stealing, they take away your phone and money if you have any. There is no way they can leave you to go scott free. (Jogoo Road, 34 years old)*

As part of their daily survival techniques in the city (see Jones and Kimari, 2019), female respondents reported offering to have sex with police to avoid the harsh reality of quarantine for those who are caught breaking the curfew rules. We were told stories of sexual assaults by police when women were apprehended during curfew times:

*Police are taking advantage of us sexually. There is a lady who was recently molested by the police. They found her during curfew time and she tried explaining that she was a sex worker and had just gone out to try her luck. They demanded to have sex with her or else they would arrest and take her to quarantine. She was so scared and since she has children, she gave in. The three of them took rounds with her and never paid. It is humiliating. (Kasarani, 22 years old)*

There have been reports of sex workers arrested during curfew hours then being quarantined in the Covid-19 facilities (Maundu, 2020). The relationship between personal safety and security in conjunction with the characteristics of chronically poor neighbourhoods in Nairobi has been long established (see Parks, 2014). What we have found here is that the police can contribute to the experiences of this group of marginalised women feeling unsafe, further extending exploitation and sexual risks (Greif et al., 2011).

## Discussion: The instability of an informal economy

The adverse impacts sex workers living and working in the informal settlements have faced during the Covid-19 crisis have resulted in dramatic changes to their livelihoods because of restrictions placed on movement and the night-time economy. The ability to earn money through the informal sex industry in Nairobi was paralysed and this research notes the following implications:

1. longer term changes to the organisation and availability of sex work as a viable income
2. increased levels of gender-based violence
3. extreme housing insecurity

First, the legacy of the government restrictions to deal with the spread of disease has drastically changed the sex work environment. Despite the loosening of movement restrictions in the daytime and the re-opening of the night-time economy for some months in 2020, there was still a reduction in the demand for sex, leaving women without an income (Wenham et al., 2020). There is a significant imbalance as the supply to sell sex far outweighs the demand to buy sex, resulting in an intensely competitive scenario which encourages women to take more risks to make cash. Irrespective of moral judgements about the rights and wrongs of selling sex, the informal economy has existed as a means to prevent impoverished women and their families (and typically female headed households) becoming destitute. The government interventions and the fear of disease have rendered the sex industry an unreliable source of survival. The case of sex workers in Kenya is further complicated by the fact that sex work is criminalised and stigmatised, an informal sector not recognised as work, making it exceedingly difficult for sex workers to benefit from any state-supported programmes.

Second, gender-based violence has been found to increase whenever there is an emergency (United Nations Development Program, 2020). It has been established as a global trend that sex workers are often victims of violence (Deering et al., 2014), particularly in settings where they and/or their clients are criminalised (Platt et al., 2018; Sanders, 2004). This is also the case in Kenya (Okal et al., 2011). The high levels of targeted violence perpetrated against sex workers has been linked with pre-existing gender inequalities and the economic dependency that follows, as well as the criminalisation of sex workers (eroding sex workers’ access to protection from authorities). The role of the police both as law enforcers and perpetrators of violence adds to the victimisation of sex workers. The Covid-19 situation has, however, introduced another layer of challenges with some women reported to be trapped with their abusers at home. In addition, if they try to work at night they are targeted by police because of the curfew restrictions. This leads authors to suggest that the urban health crisis is one which is tied up with gender-based violence (Vearey et al., 2019; Winter et al., 2020).

Third, Covid-19 has exposed new and deepening inequalities in the urban informal settlements. The unresolved land tenure and frequent demolition of houses in the informal settlements in Nairobi point to the unresolved yet important right to housing. These areas have also been excluded from major urban planning and as a result lack basic amenities, yet they are overcrowded spaces (Du et al., 2020). The social burden of the urban poor reflects the poverty and vulnerability of the sex workers highlighted in these studies. We find that the lack of reliable daily wages and exclusion from social safety nets present added danger for most of the poor urban dwellers. The desperate need for intervention for sex workers in the informal settlement requires both immediate and longer-term sustainable assistance to cushion them from both the immediate effects of Covid-19 and the longer-term legacy of the pandemic. With their working spaces, like bars and clubs, far from operating in 2021 as they had been pre-pandemic, the continued night curfews restrict their work. Sex workers are far from any normality or confidence that sex work can sustain them.

## Conclusion: Sexual citizenship and the denial of sexual labour rights

In conclusion we evaluate how our empirical data can be understood further through the theoretical lens of examining the processes of which sexual citizenship is granted to groups in the city. Hierarchies of ‘acceptable’ bodies are embedded in state discourses that transfer into ideas, policy and practice of the ‘deserving’ and the ‘undeserving’ poor (Kantola and Squires, 2004; Scoular and O’Neill, 2007). This is gendered specifically around those women who are deserving based on their compliance to gendered norms of heterosexual femininity which creates a hierarchy of those ‘others’ outside of this privileged group. Approaches to women involved in the sex industry during the pandemic crisis across the globe has often been a continuation of anti-prostitution policy, defining the moral order by delineating who is deserving of support (in this case social protections) and who is not. As Sanders (2009) has argued elsewhere, the processes through which the urban space is designed and operated as an ‘anti-sexual’ city continues these exclusionary practices based on sexual behaviour. Gendered ideals have been reproduced through the responses from governments during the pandemic with the burden of provisions for families (especially for childcare and home schooling) having fallen on women. In this research we have shown that the sex workers in Nairobi were often the head of a household, who had to shoulder extensive burdens in this time of crisis with little positive interventions from the state. Indeed, some months into the pandemic, Amnesty International (2020) noted:

*Sex workers have not only been seriously impacted by the Covid-19 pandemic, but also by governments’ emergency responses that, in many contexts, have been punitive, overbroad, and/or discriminatory. Amnesty International urges governments to take targeted action to address the disparate impact of Covid-19 on sex workers and to protect their health and other human rights*

The gendering of citizenship by the state (Cheng, 2011) is most clearly visible here with both the demolition of housing and the gender-based policing and police abuses inflicted on this group of women. These experiences have acutely demonstrated the lack of sex worker rights in a criminalised setting and the further risks that these conditions place on sex workers (Platt et al., 2018) . With such intense economic globalisation, feminisation of poverty and migration continuing to structure the lives of many women (Česnulytė, 2019; Shah, 2003) the solutions for long term economic stability and opportunity for the urban poor are far out of sight. Whilst the broader advocacy around sex worker rights was a core mission for BHESP, the everyday survival needs of sex workers during the pandemic took precedent. From our project and collaboration with our partner NGO, addressing the presenting crises of women was the primary concern to prevent the pandemic of violence, exclusion and marginalisation leading to even more fatalities.

## Notes

1 Informal settlements are commonly referred to slums which refer to highly populated urban residential area consisting mostly of closely packed, decrepit housing units in a situation of deteriorated or incomplete infrastructure, inhabited primarily by impoverished persons.

2 https://www.nation.co.ke/kenya/counties/nairobi/5-000-families-in-kariobang-homeless-after-houses-pulled-down-288950 (accessed 5 August 2020).

3 Here from BHESP staff and peers about their experiences in the pandemic 2020 https://www.youtube.com/watch?v=HsjPvsPr138 (accessed 26 July 2021).

4 https://www.africanews.com/2021/03/26/kenya-imposes-new-lockdown-what-are-the-restrictions// (accessed 26 July 2021).
